# SPME as a green sample-preparation technique for the monitoring of phytocannabinoids and endocannabinoids in complex matrices

**DOI:** 10.1016/j.jpha.2023.06.014

**Published:** 2023-06-28

**Authors:** Katarzyna Woźniczka, Paweł Konieczyński, Alina Plenis, Tomasz Bączek, Anna Roszkowska

**Affiliations:** a Department of Pharmaceutical Chemistry, Medical University of Gdańsk, Gdańsk, Poland; b Department of Analytical Chemistry, Medical University of Gdańsk, Gdańsk, Poland

**Keywords:** Phytocannabinoids, Endocannabinoids, Solid-phase microextraction, Plant material, Biological matrix

## Abstract

The endocannabinoid system (ECS), particularly its signaling pathways and ligands, has garnered considerable interest in recent years. Along with clinical work investigating the ECS’ functions, including its role in the development of neurological and inflammatory conditions, much research has focused on developing analytical protocols enabling the precise monitoring of the levels and metabolism of the most potent ECS ligands: exogenous phytocannabinoids (PCs) and endogenous cannabinoids (endocannabinoids, ECs). Solid-phase microextraction (SPME) is an advanced, non-exhaustive sample-preparation technique that facilitates the precise and efficient isolation of trace amounts of analytes, thus making it appealing for the analysis of PCs and ECs in complex matrices of plant and animal/human origin. In this paper, we review recent forensic medicine and toxicological studies wherein SPME has been applied to monitor levels of PCs and ECs in complex matrices, determine their effects on organism physiology, and assess their role in the development of several diseases.

## Highlights

• Various solid-phase microextraction protocols for isolation of phytocannabinoids in plant material and animal/human matrices.
• Applications of solid-phase microextraction for the analysis of trace levels of endocannabinoids animal and human tissues.
• Advantages and limitations of solid-phase microextraction in the analysis of phyto- and endocannabinoids in complex matrices.

## Graphical abstract

**Figure:**
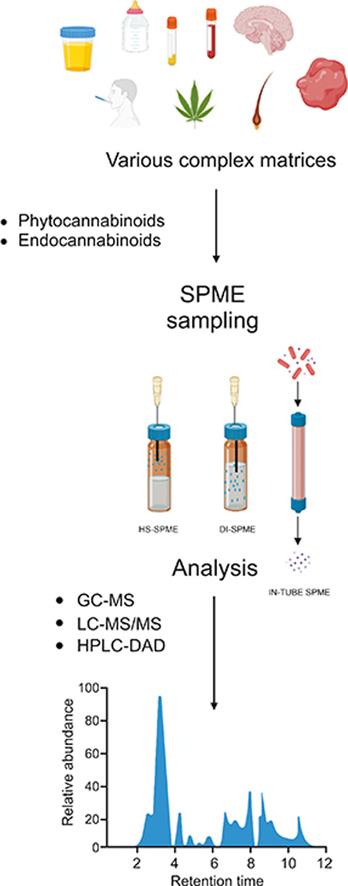

## 1 Introduction

The endocannabinoid system (ECS) is a vast neuromodulatory system that plays an important role in the function and synaptic plasticity of the central nervous system (CNS), which has drawn attention to it as a new therapeutic target in the treatment of various neurological and psychiatric conditions [1,2]. The ECS was first documented in the early 1990s as a result of work aimed at the isolation of two endogenous molecules, *N*-arachidonoyl ethanolamide (anandamide, AEA) and 2-arachidonoyl glycerol (2-AG), from pig brain samples [3,4]. In the decades since, knowledge relating to the ECS and its role in the function of the body has grown significantly due to the proliferation of research in different fields, including molecular biology, physiology, and neuropsychopharmacology, among others. The ECS is composed of multiple components, including cannabinoid receptor type 1 (CB1) and cannabinoid receptor type 2 (CB2), transient receptor potential cation channel subfamily V member 1 (TPRV1), endogenous ligands (endocannabinoids (ECs)), and enzymes that participate in the synthesis and degradation of ECs [5]. The most well-known and studied ECs are AEA and 2-AG, with findings showing that 2-AG is present in human and rodent brains at levels that are hundreds to thousands times higher compared to AEA [6]. 2-AG is the primary endogenous ligand of CB1 in the CNS, while AEA also activates other receptors, such as TRPV1 [7]. ECs are neurotransmitters that regulate functions such as energy metabolism, immune response, motor activity, and pain perception [6,8], and imbalances in their levels have been associated with the development of Parkinson's disease, epilepsy, depression, and schizophrenia [[9], [10], [11]].

In addition to endogenous regulators, the ECS is also modulated by exogenous sources, particularly phytocannabinoids (PCs), such as Δ9-tetrahydrocannabinol (Δ9-THC), cannabidiol (CBD), cannabigerol (CBG), and cannabinol (CBN), which are all natural bioactive compounds of the *Cannabis* plants. *Cannabis*-based medicines have been used in the treatment of a range of conditions, such as chronic pain [12], spastic conditions [13], and epilepsy [14], as well as to reduce the adverse effects of oncological treatment [15] and to slow the development of neurodegenerative diseases [16]. Although the use of *Cannabis*-based medicines (i.e., *Cannabis sativa* flowers for vaporization, oils, edibles) in therapies for different diseases has grown in popularity in recent years, their activity and efficiency in specific clinical conditions has not been fully proven [17]. The lack of conclusive results may be due to the fact that therapeutic effects can differ between *Cannabis* varieties and routes of administration, even though specific products may contain the same concentration of main PCs [17,18].

Hence, further knowledge about the profile of PCs in different medical *Cannabis* plants may enable a more detailed understanding of the effects of specific *Cannabis* compositions. Some authors have suggested that the term “cannabinomics” should refer to research aimed at analyzing the whole metabolome to differentiate *Cannabis* varieties and identify minor PCs [19]; conversely, others have argued that this term should refer to work focused on the simultaneous extraction and determination of ECs and PCs from different biological matrices, and the understanding of how the major and minor PCs affect the level of ECs in an organism [20]*.* Regardless, ECS has tremendous potential as a novel therapeutic target in the treatment of human diseases, especially those afflicting the CNS. Despite this potential, the interactions between PCs and the ECS are still not fully understood; thus, a global approach could help to better understand the interactions taking place within this system [5,21]. For instance, the observed interactions between PCs and ECS are complex and may have an agonistic or antagonistic impact on the ECS [11,20,22].

To precisely monitor the levels of PCs and ECs in a complex sample matrix (e.g., plant material or animal/human samples), a pre-analytical step comprising adequate sampling and sample preparation is critical to the effectiveness of the selected analytical procedure. This step typically entails the removal of undesirable matrix components from the biological samples and pre-concentrates the analytes to enhance the analytical method's sensitivity. To date, several traditional and modern sample-preparation approaches have been employed for the determination of PCs in plant [23,24] and animal/human matrices [24,25], as well as for the analysis of ECs in complex biological matrices [[26], [27], [28], [29], [30]]. However, novel microextraction technologies have been gaining interest in recent years, as they are also able to achieve the precise monitoring of these compounds and their metabolites in different matrices. Solid-phase microextraction (SPME) is one such example of a modern and rapidly developing microextraction technique. Introduced in 1989 as a single-step sample-preparation approach, SPME is based on the absorption of a target analyte from a gaseous, liquid, semi-liquid, or solid sample to a stationary phase coated onto the device. Notably, SPME can also be coupled with a variety of analytical platforms, including gas chromatography-mass spectrometry/gas chromatography-tandem mass spectrometry (GC-MS/GC-MS/MS) and liquid chromatography-mass spectrometry/liquid chromatography-tandem mass spectrometry (LC-MS/LC-MS/MS), or directly to mass spectrometry (MS) instrumentation [31]. SPME has been frequently applied for the isolation of analytes present at low levels in a variety of biological matrices. SPME offers a host of advantages, including minimal organic solvent consumption and the use of small volumes/amounts of sample material, among others [32]. In forensic and toxicological studies, SPME's ability to provide simple and fast sampling may be useful in confirming the results of on-site screening tests, particularly with respect to impaired driving or the consumption of cannabinoids in the workplace [33,34]. Nonetheless, more work remains to be done, as prior studies have indicated that false positive results for Δ9-THC might be a common issue in immunoasays (IA) testing [35]. Recently, SPME technologies have been employed to monitor PCs (mainly Δ9-THC and CBD) and their metabolites in plant and human matrices. Furthermore, SPME has also been successfully applied for the analysis of trace levels of ECs (mainly AEA and 2-AG) in animal and human tissues (plasma and brain) as part of ex vivo and in vivo studies. This paper provides an overview of the various SPME sampling protocols reported between 2012 and 2022 for the efficient and precise determination of PCs and ECs in various plant and animal/human matrices, as well as a brief summary of other extraction techniques that have been applied to this end. Furthermore, we consider the advantages and limitations of studies utilizing various traditional and SPME-based approaches, and outline future perspectives in the determination of PCs and ECs in complex matrices.

## 2 SPME—general information

SPME has been widely applied for the targeted and untargeted analysis of low-molecular-weight compounds, such as drugs, environmental pollutants, food additives, and endogenous compounds (metabolites) [32,36]. SPME's excellent diversity with respect to device geometry (e.g., coated fiber, thin-film, mesh, blade, in-tube SPME (IT-SPME)) and extraction phases makes it ideal for bioapplications ranging from the in vitro sampling of cell cultures to in vivo determination performed on living organisms [37]. SPME sampling of volatile and semi-volatile compounds is performed in headspace (HS) mode with a variety of coatings (extraction phases) that can be tailored to the physico-chemical properties of the targeted compounds [38,39]. Furthermore, the introduction of new coating particles, such as commercially available octadecyl (C_18_) and divinylbenzene (DVB) and in-house-made hydrophilic-lipophilic balanced (HLB), strong cation exchange (SCX), and several mixed-mode coatings (e.g., octyl-strong cation exchange (C_8_-SCX) have extended SPME's applications to include direct immersion (DI) mode sampling of non-volatile analytes with various polarities. Recently, researchers have directly coupled SPME devices to MS via nano-electrospray ionization (ESI), microfluidic open interface (MOI), and coated blade spray (CBS), which has enabled the fast and reproducible analysis of small sample volumes without chromatographic separation, and thus, higher enrichment factors and enhanced sensitivity. Furthermore, the use of IT-SPME allows the on-line determination of small amounts of biological sample [40,41], while new trends towards the automation or semi-automation of whole extraction procedures (i.e., preconditioning, extraction, washing, and desorption steps) and the potential for on-site sampling have made it possible to apply SPME for clinical and toxicological purposes [[42], [43], [44]]. Furthermore, the coating's ability to restrict the access of macromolecules to the sorbent while allowing selected small molecules to penetrate enables the quenching of the extracted compounds, which protects them from subsequent degradation and/or enzymatic transformation [31,32]. These foundational properties facilitate the use of SPME for the determination of a wide range of compounds, especially labile or short-lived molecules. In addition, SPME sampling does not disturb the biochemical equilibrium in the system under study, a feature that has been well-demonstrated in various metabolomics and drug monitoring studies performed directly in living systems (in vivo SPME) [31,45,46]. An overview of recent progress in the fundamentals of SPME, the challenges and practical implementation of this technique in different areas of science, including in vivo analysis of living systems as part of biomarker discovery studies, toxicological analysis, and environmental monitoring has been presented in several review articles and book chapters [31,47,48]. Recently, SPME has been proposed for the isolation of PCs and ECs from different sample matrices, including plant material [[49], [50], [51]] and various biological samples, such as plasma and brain tissue [27,40,[52], [53], [54]].

## 3 PCs—chemical structures, metabolism, and mechanisms of action in humans

**Fig. 1:**
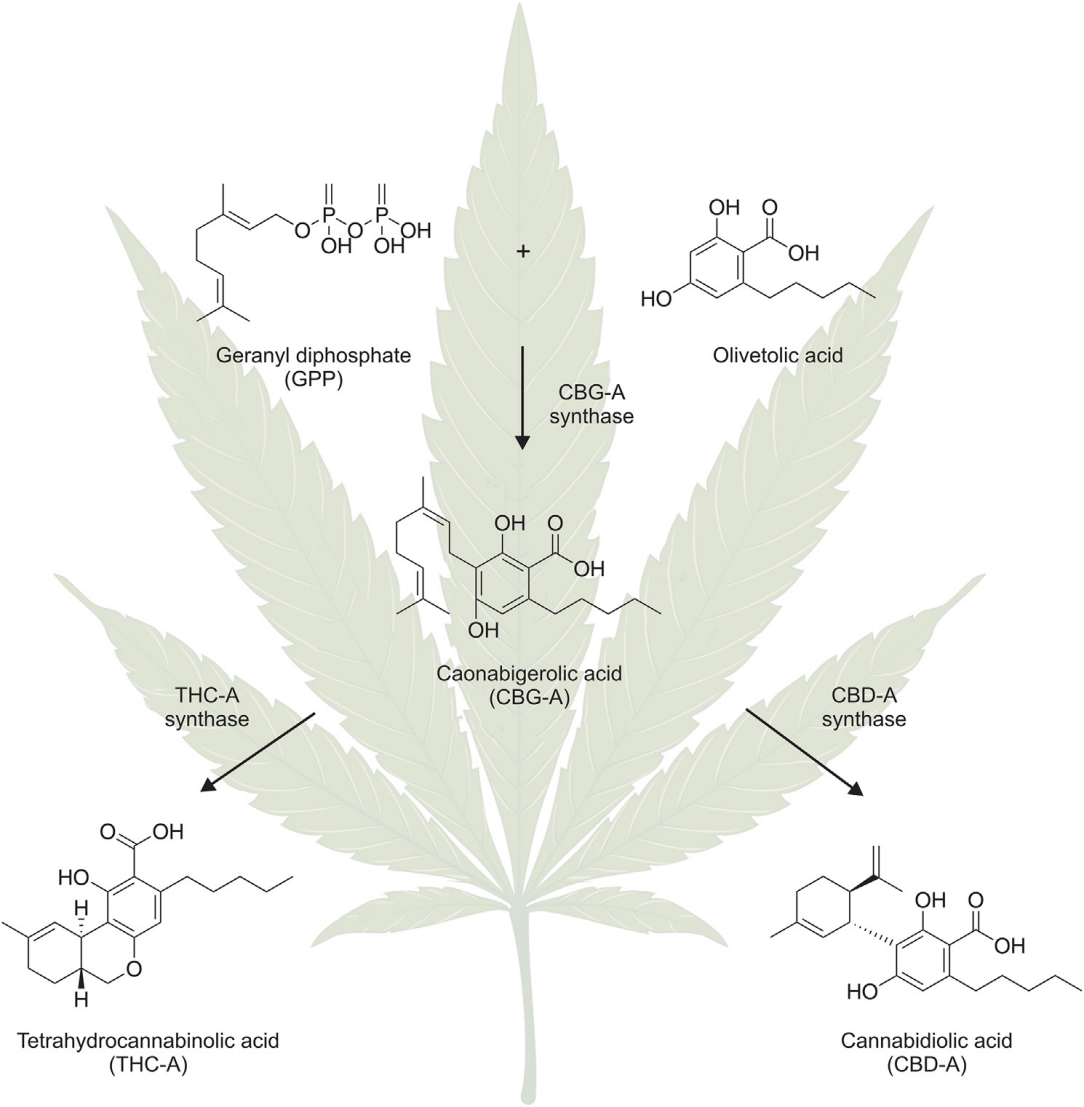
A simplified pathway biosynthesis of tetrahydrocannabinolic acid (THC-A) and cannabidiolic acid (CBD-A) in *Cannabis* plants. Figure is created with BioRender.com.

*Cannabis sativa* L. is a dioecious plant that has been used for medical purposes in humans since ancient times [55]. However, investigations into *Cannabis sativa'*s pharmacological properties and bioactive components, including PCs, have been limited due to its illegal status in many countries [56]. PCs are natural bioactive compounds characterized by terpenophenolic structures. To the present, more than 140 cannabinoid compounds have been isolated from *Cannabis sativa* and divided into 11 subclasses; however, the compounds from 6 of these subclasses are probably the products of the decomposition of main cannabinoids due to oxidation or poor storage conditions [18]. PCs are synthesized in structures known as glandular trichomes, which are present in high densities on the female flowers of the *Cannabis* plant where they produce resins composed of cannabinoids, terpenes, and flavonoids [57]. While PCs are synthesized in acidic form (e.g., Δ9-Tetrahydrocannabinolic acid (THC-A), cannabidiolic acid (CBD-A), cannabigerolic acid (CBG-A)), heating them initializes a decarboxylation process whereby the carboxyl group is disconnected from the chemical structures of particular cannabinoids to form neutral, more pharmacologically potent compounds that can easily penetrate the blood-brain-barrier [58]. Decarboxylation occurs spontaneously when *Cannabis* flowers are vaporized or smoked, but it also plays a role in magistral drug preparation [13,56]. The acidic forms of the main cannabinoids in plants are formed from olivetolic acid and geranyl diphosphate (GPP) as a result of enzymatic reactions. GPP is also a substrate in the synthesis of monoterpenes [59]. The precursor for the majority of PCs is CBG-A, which is transformed into PCs belonging to various subclasses as a result of enzymatic reactions (Fig. 1). The specific composition and proportions of cannabinoids, terpenoids, and flavonoids are responsible for the so-called “entourage effect,” which refers to the pharmacological effect of the whole plant. Notably, findings have shown that this effect is not present in chemically synthesized cannabinoids or in individual substances isolated from the resin of female flowers of the *Cannabis* plant [60].

The most studied and frequently applied PCs in pharmacotherapy are Δ9-THC, CBD, and CBG. PCs are highly lipophilic compounds with logP values greater than 5 for both their neutral and acidic forms [58]. Acidic forms of PCs possess a pKa of around 2.9 and exist in ionized form in physiological pHs, which explains their poor penetration to the CNS [58]. As already mentioned, PCs primarily act on the ECS in humans, specifically on the CB1 and CB2 receptors, and also on the orphan G receptors, TRPV1, peroxisome proliferator activated receptors (PPARs), serotonin receptors (5-HT), and opioid receptors [22,61]. PCs differ in their affinity for specific receptors [22]. For instance, Δ9-THC is a partial agonist of the CB1 and CB2 receptors and an antagonist of the 5-HT_3_ receptors. Conversely, CBD is an antagonist/allosteric modulator for the CB1 receptors and an antagonist for the CB2 receptors. This variance explains the modulatory effect of Δ9-THC activity. Furthermore, CBD also inhibits AEA reuptake. Both Δ9-THC and CBD are allosteric modulators for opioid receptors μ and δ [22]. On the other hand, CBG is a low affinity partial agonist for the CB1 and CB2 receptors and inhibits AEA reuptake [22]. Although there is limited information relating to the pharmacokinetics and modes of action of acidic PCs, animal-model studies have indicated that CBD-A possesses anti-inflammatory, and antihyperalgesic properties, while in vitro studies have shown that THC-A possesses anti-inflammatory properties [58]. Overall, it is difficult to determine PCs’ precise mechanism of action for single components, as they tend to foster the group-like interaction/modulation of receptors, rather than affecting/modulating a single receptor.

**Fig. 2:**
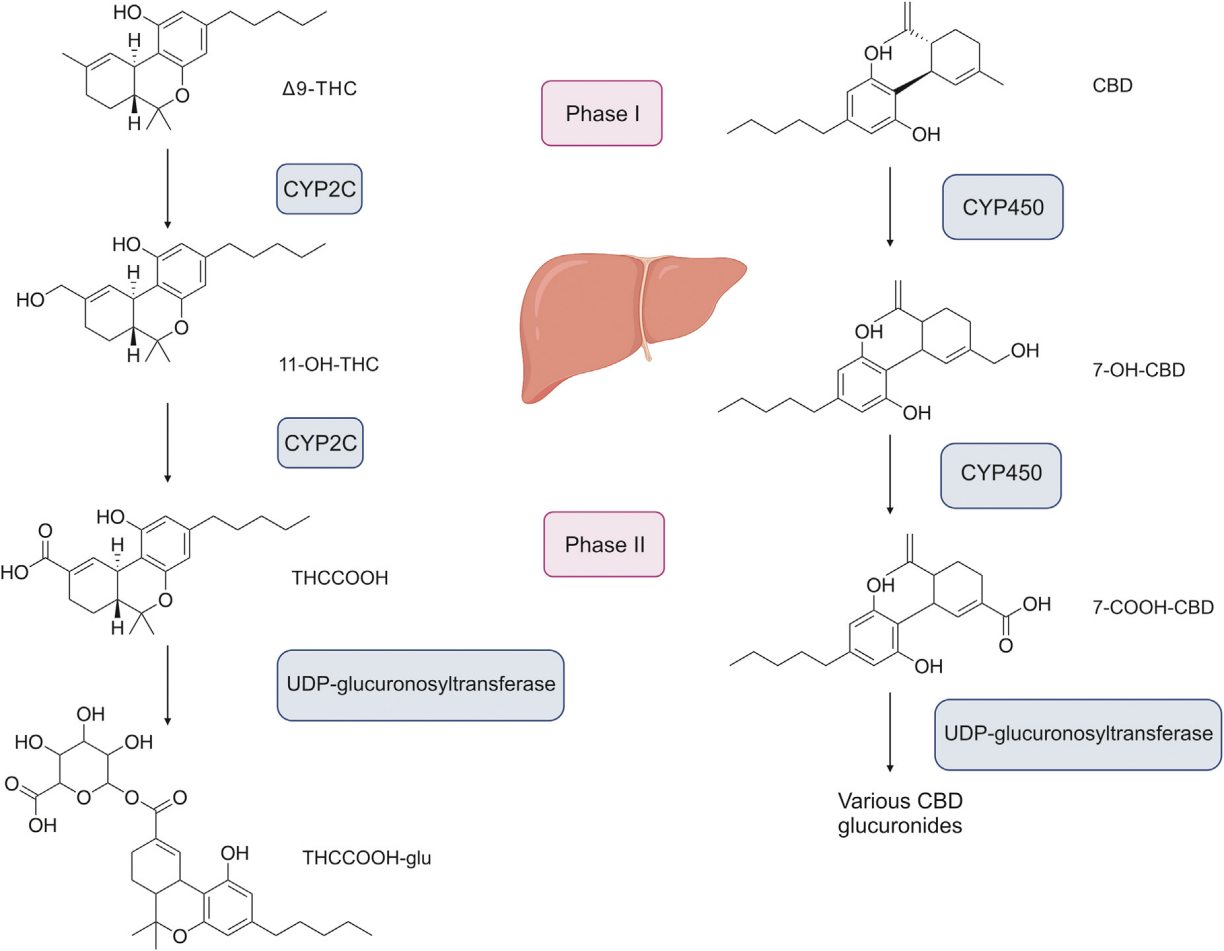
A simplified pathway of Δ9-THC and CBD metabolism in humans. Δ9-THC: Δ9-tetrahydrocannabinol; 11-OH-THC: 11- hydroxy-tetrahydrocannabinol; THCCOOH: 11-nor-9-carboxy-tetrahydrocannabinol; THCCOOH-glu: 11-nor-Δ9-tetrahydrocannabinol-9-carboxylic acid glucuronide; CBD: cannabidiol; 7-OH-CBD: 7-hydroxycannabidiol; 7-COOH-CBD: 7-carboxy cannabidiol. Figure is created with BioRender.com.

The main PCs (i.e., Δ9-THC and CBD) are hepatically metabolized by CYP450 isoenzymes (Fig. 2). CYP2C9 plays a major role in the phase I metabolism of Δ9-THC, as it is responsible for converting it to its primary metabolite, 11-hydroxy-Δ9-tetrahydrocannabinol (THC-OH), which possesses psychotropic properties [5,62]. Further oxidation leads to the formation of 11-nor-9-carboxy-Δ9-tetrahydrocannabinol (THCCOOH). The phase II metabolism of PCs includes glucuronidation, which results in the formation of the water-soluble metabolite, 11-nor-Δ9-tetrahydrocannabinol-9-carboxylic acid glucuronide (THCCOOH-glu). This metabolite is more amenable to elimination via the urine [5]. However, little is known about the metabolism of CBD. At present, it is known that 7-carboxy-cannabidiol (7-COOH-CBD) and its derivates are main metabolites formed in the phase I hepatic metabolism of CBD, and that CBD glucuronide is a major product of its phase II metabolism [62].

## 4 Determination of PCs in complex matrices

The number of protocols for the extraction and determination of PCs in plant and human matrices has grown significantly over the last 20 years [63]. Most studies using plant material have focused on monitoring the content of particular cannabinoids, which are strongly dependent on growing conditions. From an analytical perspective, several reports have highlighted instability issues and the need to apply mild conditions when designing protocols for the determination of PCs [49]. With regards to instrumental analysis, GC-MS has long been recognized as the “gold standard” for the analysis of PCs in forensic studies. However, several studies have noted that cannabinoids are thermally labile in both their acidic and neutral forms, which raises the possibility that GC-based analytical methods may result in their degradation [18,49]. On the other hand, LC-based methods allow the accurate proﬁling of PCs (including distinguishing acidic and neutral forms), as they do not require thermal desorption or analyte derivatization, which is often employed in GC-based determination of these compounds [64]. Furthermore, other instrumental methods can also be employed for the analysis of PCs in plant material, including supercritical ﬂuid chromatography (SFC), infrared spectroscopy (IR), and Raman spectroscopy [63]. Nonetheless, LC-MS/MS-based methods are the dominant approaches used in contemporary analyses of PCs to avoid degradation due to their thermolability [18,23,24]. For instance, Abd-Elsalam et al. [65] applied LC-MS/MS to quantitate PCs and their metabolites in various biological matrices, such as blood and blood derivatives, urine, oral fluid (OF), and hair samples. Conversely, Citti et al. [24] documented a wide range of methods for extracting and detecting PCs in plant and biological matrices. In Europe, the official methodology for analyzing pharmacological plant material is based on the German Pharmacopeia, which prescribes the use of high-performance liquid chromatography coupled with ultraviolet detection (HPLC-UV) for the quantification of acidic and neutral forms of CBD and Δ9-THC, as well as for CBN as a decomposition product of Δ9-THC [23,66].

Among extraction techniques applied in the determination of PCs in plant material, solid-liquid extraction is the most widely used one, although supercritical fluid extraction (SFE) with ethanol as a co-solvent is becoming more popular for the simultaneous extraction of terpenes and cannabinoids, especially for consumptive purposes [24]. Notably, the *Cannabis extractum normatum* monograph in the German Pharmacopeia describes SFE with carbon dioxide as the preferred extraction method of PCs [67]. In addition, sample preparation techniques based on dynamic maceration and ethanol extraction have been frequently used for identification and quantification of neutral and acidic forms of PCs in various strains of *Cannabis* plants [18,68]. Ethanolic extraction is also a part of the sample-preparation protocol described in the *Cannabis flos* monograph of the German Pharmacopeia [66]. An interesting review of the different sample-preparation approaches (solvent- and sorbent-based) and GC- and LC-based analytical methodologies that have been employed for the analysis of cannabinoids and terpenes in plant materials can be found in a study by Micalizzi et al. [64].

In the case of biological matrices such as plasma and urine, liquid-liquid extraction (LLE) [69,70] and solid-phase extraction (SPE) are the most widely used extraction techniques in the determination of PCs. Also, simple protein precipitation (PPt) or the use of combined techniques, such as PPt and supported liquid extraction (SLE) or PPt and SPE, has been applied for the isolation of selected PCs from complex matrices [24,58,[71], [72], [73], [74]]. In addition, chemical and enzymatic hydrolysis have been applied to release ester and ether glucuronide forms of cannabinoids prior to the extraction of PCs from urine samples [75,76]. The first applications of different microextraction techniques, including SPME, for the analysis of PCs in human matrices were reviewed in a paper by Jain et al. [77] Recently, the use of sorbent-based microsampling approaches other than SPME (i.e., microextraction by packed sorbent (MEPS)) for the determination of PCs in human biofluids has been reported [78,79]. However, MEPS-based methods were employed solely for extractions from biological fluids because, unlike SPME, they cannot be applied for the analysis of PCs in solid tissues, such as plant matter or hair samples.

### 4.1 SPME in determination of PCs in plants and animal/human matrices

SPME with various modifications has been employed to extract PCs from complex matrices of plant and human origin as a part of forensic studies and doping control efforts, as well as to characterize the composition of cannabinoids in plant material. Since PCs are semi-volatile compounds, they can be isolated using either HS- or DI-SPME mode. As highlighted in several studies, high temperatures cause the decarboxylation of acidic forms of PCs, and they can also contribute to the decomposition of their neutral forms [49]. Hence, ensuring that sample handling, extraction, and determination all take place under precise and controlled conditions is critical to obtaining reliable results concerning the levels of PCs in plant and human matrices.

#### 4.1.1 The application of SPME in the extraction of PCs from plant material

Numerous studies have demonstrated SPME's utility for the determination of trace amounts of PCs in plant matter, including in small amounts of sample. For instance, IT-SPME was coupled with a miniaturized chromatographic system (nanoLC) to determine Δ9-THC, CBD, and CBN in dried plant material (<1 mg) on the surfaces of plastic bags, aluminum foil, office paper, and skin [50]. The suspected drug samples were collected with cotton swabs. Prior to SPME, the analytes were extracted from swabs with the use of methanol in an ultrasonic bath for 15 min. Next, the analytes were subjected to IT-SPME utilizing 95% polydimethyl siloxane, 5% polydiphenylsiloxane (TBR-5) column. Δ9-THC concentrations were in the 41–99 ng/mL ranges, which constituted 0.6%–2.8% of Δ9-THC in the samples. In other work, Calvi et al. [80] applied a modified HS-SPME techniques to extract terpenes from *Cannabis* oils and inflorescences, while Ternelli et al. [81] used it to extract terpenes from *Cannabis* oils. In these works, the authors extracted PCs with the use of organic solvents and analyzed them via LC-based methods; however, modified HS-SPME could be also implemented for the extraction of PCs. In another study, heated HS-SPME (HHS-SPME) was coupled with GC-MS for the determination of 14 *Cannabis sativa* samples with known levels of Δ9-THC and CBD [51]. The developed method was part of an analytical platform that incorporated a machine learning algorithm to differentiate *Cannabis sativa* varieties. Notably, the high temperatures applied during HS extraction precluded the detection of acidic forms of PCs. In a different work, regular and vacuum HS-SPME (Reg-HS-SPME and Vac-HS-SPME) protocols were coupled with GC-MS for the simultaneous determination of CBD, cannabichromene (CBC), as a decomposition product of CBD, and terpenoids in dried *Cannabis* inflorescences from CBD-rich chemotypes [49]. In this study, the authors tested two SPME fiber coatings: overcoated polydimethylsiloxane/divinylbenzene (PDMS-DVB) and divinylbenzene/carboxen/polydimethylsiloxane (DVB-CAR-PDMS), at different extraction temperatures (80, 90, and 150 °C), and sampling time (5, 15, and 30 min). Ultimately, the PDMS-DVB coating was chosen, as it provided higher recoveries of CBD than the DVB-CAR-PDMS coating. Furthermore, the findings also indicated that acceptable recoveries could be attained for cannabinoids and terpenoids at 90 °C, and that the applied temperature conditions under reduced pressure were 60 times more efficient for the extraction of CBD. The authors demonstrated that, at 150 °C, CBD degrades to CBC and probably to Δ9-THC and Δ8-THC in tested conditions. Since cannabinoids are semi-volatile compounds, reducing the pressure in the sampling vial increased the mass transfer into the HS zone; this phenomenon was used in Vac-HS-SPME. However, the authors noted that the partial decarboxylation of acidic forms of cannabinoids may occur at lower temperatures, and thus, the obtained result may be inadequate. The developed Vac-HS-SPME coupled to GC-MS method could be considered as greener alternative protocol for analysis of PCs and terpenes in plant material.

So far, the SPME studies applied in the determination of PCs in *Cannabis* plants were based on IT-SPME or HS-SPME sampling. In the first approach, additional sample handling was necessary prior to online IT-SPME; however, the proposed protocol was fully validated for quantifying PCs in plant material. In the case of HS mode, the isolation of PCs facilitated only semi-quantitative analysis. This type of extraction of PCs from complex matrices requires careful adjustment of extraction temperature and time so as to avoid the risk of degradation of acidic and neutral forms of cannabinoids.

#### 4.1.2 The application of SPME for the extraction of PCs from human matrices

**Fig. 3:**
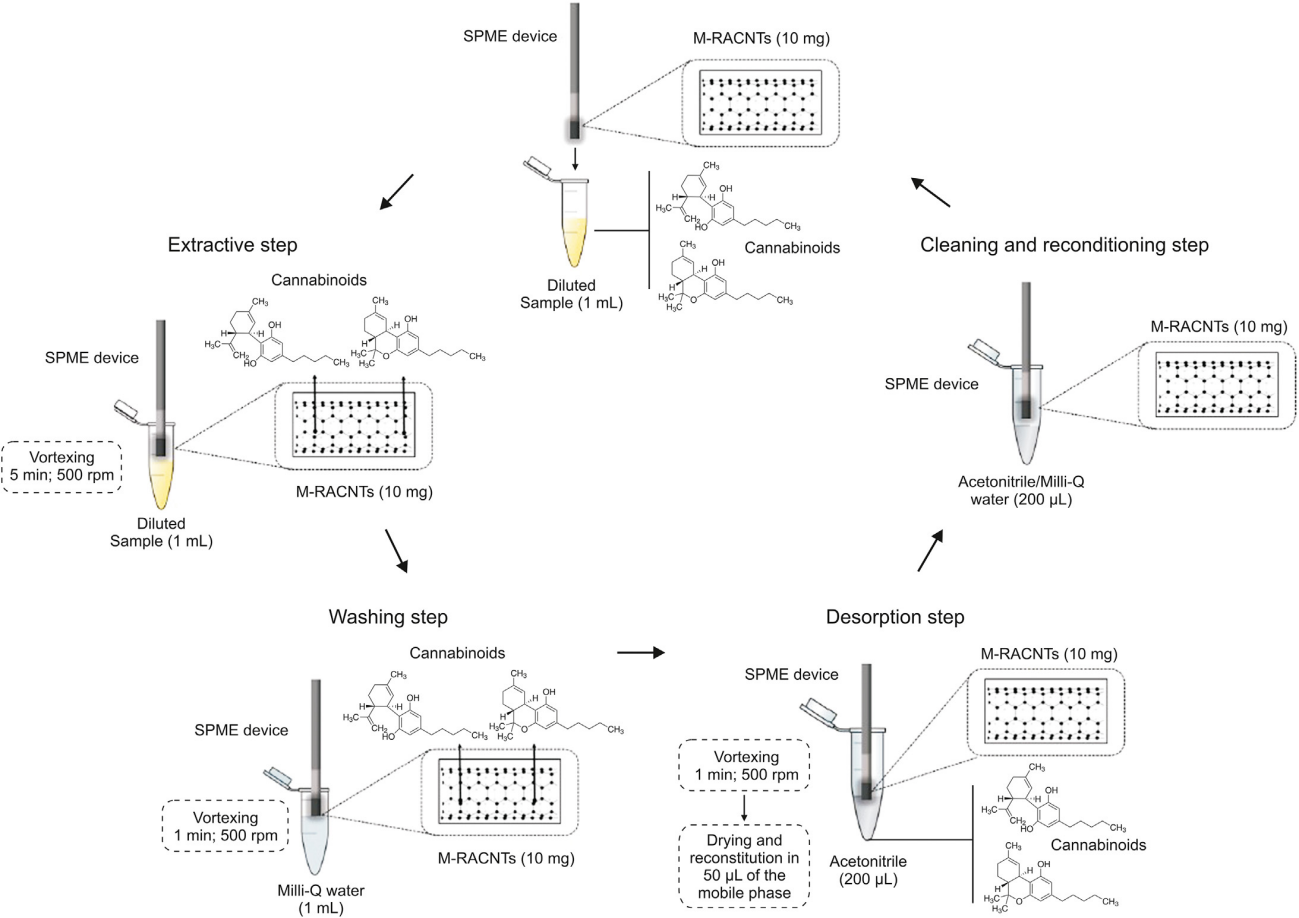
Analytical workflow solid-phase microextraction (SPME)-based method. Reprinted with permission from Ref. [54].

Since PCs and their metabolites are characterized by high plasma protein binding (∼97%) [82], the application of SPME will yield information about the free, pharmacologically active fraction of these compounds [32]. A number of researchers have combined SPME-based protocols with LC-MS/MS for the isolation and determination of PCs in plasma samples [40,44,83]. Reyes-Garcés et al. [84] proposed a typical SPME workflow for the quantification of 25 doping substances and their metabolites, including the Δ9-THC metabolites, THCCOOH-glu (polar) and THCCOOH (non-polar), in human plasma samples. To enable the simultaneous determination of multiple samples, an automated Concept 96-blade SPME system was used. In addition, SPME's open-bed geometry facilitated the rapid isolation of compounds from complex biological matrices without any clogging issues, thus eliminating the need for a PPt step prior to the sample preparation. Although this protocol was not employed in clinical or forensic applications, the results highlighted several practical findings related to the extraction conditions for multiple compounds with different polarities (logPs ranging from −2 to 6.8) and protein binding affinities. Of the sorbents tested during the method-optimization stage (polystyrene-divinylbenzene (PS-DVB), HLB, C_18_), the biocompatible HLB coating provided the best coverage for all analyzed compounds (polar and nonpolar) and the lowest carry-over effects; thus, this coating was selected for use in subsequent experiments. However, it is worth noting that the C_18_ fiber provided better extraction efficiency for nonpolar compounds, such as THCCOOH. The authors observed that the use of the increase of only 5 °C in the extraction temperature led to significant increase in the amounts extracted for some of the studied drugs, including THCCOOH and THCCOOH-glu, and ultimately 30 °C was selected as the extraction temperature. In addition, protein binding significantly affected the extraction time for plasma samples such that substances with high protein binding properties experienced difficulties in reaching the equilibrium [44]. PCs with high protein binding properties also have high logP values, and this information can be significant in optimizing method development for PCs analysis. Elsewhere, IT-SPME was proposed for the quantification of CBD and Δ9-THC in plasma samples collected from healthy volunteers who had been treated with CBD (300 mg) [40]. In this study, the authors synthesized a molecularly imprinted monolithic capillary for the isolation of both analytes; however, the on-line nature of the developed method necessitated the introduction of a sample-pretreatment step (i.e., PPt) to prevent capillary clogging. Methacrylic acid was chosen as the monomer for imprinting due to the interactions between its hydrogen bonds and the template, and a dummy template (hydrogenated CBD) was employed to avoid “template bleeding” and to minimize the risk of false-positive results [84]. The resultant IT-SPME-LC-MS/MS method was applied for the monitoring of CBD levels in plasma, and the concentrations of this PC in plasma samples ranged from 35.9 to 213.5 ng/mL. The results also revealed that no Δ9-THC was detected in the plasma samples, which may indicate that CBD does not metabolize to Δ9-THC. The SPME was also applied to quantify CBD and Δ9-THC in plasma samples from two groups of volunteers: chronic cannabis smokers and volunteers taking CBD capsules [54]. To isolate both analytes, the authors used a sorbent consisting of magnetic-restricted access carbon nanotubes (M-RACNTs). To this end, Fe_3_O_4_ magnetic nanoparticles (MNPs) were incorporated into carbon nanotubes and coated with bovine serum (BS). Since negatively charged BS and human plasma proteins create space for analyte bonding at pH values higher than the isoelectric protein point, a PPt step was not necessary. Based on their results, the authors concluded that the sorption equilibrium time and volume of desorption solvent significantly influence analyte recovery when using SPME (Fig. 3) [54]. The concentrations of Δ9-THC measured in cannabis smokers ranged from 14.92 ng/mL to 21.13 ng/mL, while the concentrations of CBD measured in volunteers after the intake of CBD capsules ranged from 10.14 ng/mL to 17.34 ng/mL.

Easy and non-invasive collection make OF a useful matrix for detecting the presence of PCs in roadside screening tests and anti-doping tests [33,42]. However, OF is not an ideal matrix for the therapeutic drug monitoring (TDM) of PC levels in medical cannabis users, as the concentrations of these compounds in OF do not reflect blood concentrations, which means information about their activity may be imprecise. One reason for this discrepancy may be the local absorption of PCs in the oral cavity [85]. Nevertheless, Anzillotti et al. [33] proposed an SPME-GC-MS protocol for the quantification of 3 PCs (Δ9-THC, CBD, CBN) in OF samples collected with commercially available OF collection devices (DDS and DCD5000) during a roadside inspection after preliminary positive Δ9-THC screening tests. The proposed method facilitated the quantification of PCs in small sample volumes with no initial sample pretreatment and revealed that 28 of the samples had produced false-positive for Δ9-THC in the preliminary roadside tests. Additionally, the proposed method was also able to detect the presence of CBD and CBN in the samples (Table 1). Furthermore, the SPME-GC-MS method provided lower limit of quantification (LOQ) values (especially for CBD and CBN) compared to a concurrently developed ultra-high-performance liquid chromatography-tandem mass spectrometry (UHPLC-MS/MS) method. It was observed that Δ9-THC was stable in real samples both in DDS buffer and in neat OF, and could be quantified with acceptable reproducibility and accuracy criteria. The mean concentration for Δ9-THC found was 58 ng/mL, while CBD had a mean value of 38 ng/mL. The authors also reported that the concentrations of CBN in OF ranged from limit of detection (LOD) to 375 ng/mL. In another work, an analytical method based on SPME-LC-MS enabled the simultaneous determination of 49 substances and their metabolites (including THCCOOH, CBD, and CBN), including a selection of substances prohibited by World Anti-Doping Agency (WADA) [42]. The findings of this study showed that the proposed SPME method produced cleaner extracts compared to LLE in terms of absolute matrix effects. However, despite acceptable validation parameters for cannabinoids, the developed protocol has not been used in practice. In a different study, HS-SPME was combined with GC-MS analysis for the detection of 7 PCs: Δ9-THC, CBD, CBN, Δ8-THC, CBC, CBG and tetrahydrocannabivarin (THCV), in different buccal swabs [86]. The purpose of this study was to determine whether HS-SPME could be used to analyze air-dried buccal swabs obtained in the collection of forensic evidence. In considering this study, it is important to note that the authors did not use real samples; rather, they used buccal swabs that had been spiked with a cannabinoid mixture. The authors tested two sample-preparation techniques: one without derivatization and one with in situ (in vial) derivatization with *N*-methyl-*N*-(trimethylsilyl)trifluoroacetamide (MSTFA) during HS-SPME. Although derivatization increased the abundance and improved the peak shapes of Δ9-THC, acidic forms of PCs (THC-A, CBD-A, and CBG-A) were still not detected by the GC-MS approach due to their thermally labile nature. Another research group applied DI-SPME followed by GC-MS to determine PCs (Δ9-THC, CBD, CBN) and synthetic cannabinoids in OF [34]. To determine the optimal coating, preliminary experiments were conducted in HS-SPME and DI-SPME modes using 85 μm polyacrylate (PA), 100 μm PDMS, 7 μm PDMS, and 65 μm PDMS/DVB coatings. The results of these tests indicated that the best extraction efficiency was provided by the 100 μm PDMS fibers in DI-SPME mode. Although both modes enabled the detection of PCs within acceptable parameters, DI-SPME proved to be superior in the determination of synthetic cannabinoids. Ultimately, a DI-SPME-GC-MS protocol was employed to analyze 106 authentic OF samples. The presence of PCs was detected in four of the analyzed samples, but no synthetic cannabinoids were detected in any of the samples (Table 1). In the positive samples, the concentration of Δ9-THC ranged from 10 to 655.2 ng/mL, CBD from 4.5 to 15.3 ng/mL, and from <LOQ to 66.2 ng for CBN ng/mL.

**Table 1:** The overview of analytical approaches utilizing SPME-based techniques along with instrumental methods applied in the analysis of phytocannabinoids (PCs) in plant material and biological matrices.

Analyte(s)	Sample type and volume	Sample preparation		Instrumental analysis		Internal standard(s)	LOD/LOQ	Linearity		Application	Refs.
		Pretreatment	Extraction	Chromatographic separation	Detection						
Δ9-THC, CBD, CBN	OF (100 μL)	Samples collected with commercial OF collection devices: Concateno DDS (OF was diluted in preservative buffer.) and Draeger DCD5000 (OF was diluted in 100 μL of mobile phase A.)	SPME with 100 μm PDMS coating, extraction: 30 min, desorption: 2 min, in GC injection port at 270 °C	GC-MS, Helium as a carrier gas flow rate 1 mL/min	Column J&W DB-5 (5% phenylmethylsilicone) capiilary column (15 m × 0.2 mm, 0.33 μm)	Δ9-THC-d3	LOD: 0.5 ng/mL for Δ9-THC, 2 ng/mL for CBN, 2 ng/mL for CBD	2–200 ng/mL for Δ9-THC LOQ (not provided)–200 ng/mL for CBD and CBN	Analysis of 70 real OF samples from roadside tests		[33]
Δ9-THC, CBD, CBN and 9 synthetic cannabinoids	OF (1 mL)	Centrifugation for 5 min; 1 mL of supernatant was collected.	DI-SPME with 100 μm PDMS coating, extraction: 15min, desorption: 2 min, in GC injection port at 270 °C	GC-MS, Helium as a carrier gas at a flow rate 1 mL/min	Column DB-5 (30 m × 0.25 mm, 0.25 μm)	Δ9-THC-d3	LOD: 1 ng/mL, LOQ: 1 ng/mL for Δ9-THC; LOD: 1 ng/mL, LOQ: 10 ng/mL for CBN; LOD: 5 ng/mL, LOQ: 10 ng/mL for CBD	LOQ–1000 ng/mL	Analysis of 106 OF samples collected from volunteers		[34]
Δ9-THC and 10 other common abuse substances	Human sweat	Samples were collected with Drugwipe® pad; - pad was then acidified with 1 M HCl for 60 min at 60 °C, and extracted acid layer was mixed with 200 mg K_2_CO_3_; - THC extraction: after first extraction, the pad was treated with 1 mL of 1 M NaOH and 0.5 g of NaCl.	HS-SPME with 100 μm PDMS coating, - first extraction: 10 min at 90 °C, and then the fiber was exposed for 3 min at 90 °C in the HS of another vial containing 5 μL of acetic anhydride for derivatization; - second extraction (THC): 30 min at 150 °C, and then the fiber was exposed for 10 min at 90 °C in another vial containing 5 μL of MSTFA for derivatization; thermal desorption: 3 min in GC injection port at 250 °C	GC-MS, Helium as the carrier gas at flow rate 1.0 mL/min	Fused capillary column (5% pH ME Siloxane) (12.5 m × 0.20 mm, 0.33 μm)	Δ8-THC	LOD: 0.09 ng/pad, LOQ: 0.27 ng/pad	LOQ–50 ng/pad	Analysis of sweat samples collected from 66 suspected drugged drivers stopped during roadside controls		[35]
Δ9-THC, CBD	Human plasma (300 μL)	PPt with 600 μL of ACN; supernatant was dried and then reconstituted with 50 μL of 5 mM ammonium acetate (pH 3.0) aqueous solution.	IT- SPME with molecularly imprinted capillary column (10.0 cm × 0.53 mm) with hydrogenated cannabidiol as a dummy template; 10 μL of sample solution was flown through capillary with ACN as a sorption solvent and 5 mM ammonium formate solution as a washing solvent; flow rate: 0.02 mL/min	UHPLC-MS/MS gradient mode, phase A: 5 mM ammonium acetate, phase B: ACN with 0.1% FA, flow rate: 0.3 mL/min at 40 °C	Kinetex C_18_ column (100 mm × 2.1 mm, 1.7 μm)	CBD-d3, Δ9-THC-d3	LLOQ: 10 ng/mL for Δ9-THC and CBD	10–300 ng/mL	Analysis of plasma samples collected from 14 volunteers treated with 300 mg CBD capsules.		[40]
Δ9-THC, THCV, CBD, CBN, THC-OH, THCCOOH, THCCOOH-glu	Human urine (250 μL)	PPt with 500 μL of ACN; 500 μL of supernatant was used for analysis.	Column switching IT- SPME packed with LichroPrep RP-18 sorbent in stainless tube (508 μm i.d.); 50 μL sample solution was percolated through capillary using 22% ACN aqueous solution; flow rate: 0.15 mL/min, extraction: 1 min; analytes were washed out with gradient.	LC-MS/MS Gradient elution, phase A: 0.1% FA, phase B: ACN with 0.1% FA, flow rate: 0.3 mL/min at 40 °C	Poroshell 120 EC - C_18_ column (2.1 mm × 100 mm, 2.7 μm)	THC-d3, CBN-d3, CBD-d3, THC–OH–d3, THCCOOH-d3, THCCOOH-glu-d3	LOQ: 10 ng/mL for Δ9-THC, THCV, CBD, CBN, THC-OH, THCCOOH; LOQ: 25 ng/mL for (THCCOOH-glu)	10–160 ng/mL for Δ9-THC, THCV, CBD, CBN, THC-OH, THCCOOH, 25–1,000 ng/mL for THCCOOH-glu	Analysis of 20 urine samples obtained from volunteers (*Cannabis* users)		[41]
THCCOOH, CBD, CBN and other 46 substances and their metabolites	OF (1.2 mL)	–	TF-SPME fiber with C_18_ sorbent, extraction: 75 min, desorption: 60 min, with 1.2 mL ACN:MeOH:H_2_O (40:40:20, *V/V/V*)+0,1%FA	LC-MS/MS Gradient mode, phase A: water:ACN:FA (89.9:10:0.1,*V/V/V*), phase B: ACN:FA (99.9:0.1,*V/V/V*), flow rate: 0.3 mL/min at 25 °C	Kinetex PFP column (100 mm × 2.1 mm, 2.6 μm)	–	LOQ: 0.07 ng/mL for THCCOOH and CBN, 0.09 ng/mL for CBD	–	Analysis of samples spiked with the analytes using fully automated Concept 96 device		[42]
Δ9-THC, CBD, CBN, THCCOOH and 106 other doping substances and their metabolites	Human urine (9 mL)	1 mL of 2 M PBS spiked with ISs was added to 9 mL of urine sample; 1.2 mL of buffered urine was used for further analysis.	TF-SPME fiber with C_18_ sorbent, extraction time: 75 min, desorption: time 60 min, desorption solvent: 1.2 mL ACN:MeOH:H_2_O (40:40:20, *V/V/V*)+0,1%FA	LC-MS/MS gradient elution, mobile phase A: 0.1% FA, mobile phase B: ACN with 0.1% FA, mobile phase C: MeOH with 0.1% FA at 30 °C	Kinetex PFP column (100 mm × 2.1 mm, 1.7 μm) with guard filter (Security Guard ULTRA Cartridges UHPLC PFP for 2.1 mm)	CBD-d3 and 9 other deuterated compounds	LOQ: 5 ng/mL	–	Analysis of urine samples spiked with analytes using fully automated Concept 96 device		[43]
THCCOOH, THCCOOH-glu and 23 other compounds with different polarities	Human plasma (1,080 μL)	Plasma samples was mixed with 120 μL PBS and agitated for 30 min, and then preincubated in 30 °C for 30 min.	TF-SPME with HLB coating, extraction: 90 min at 30 °C, desorption: 20 min with 1,200 μL solution of MeOH:ACN (4:1, *V/V*) with 0.1% FA;	UHPLC-MS/MS Gradient mode, phase A: 0.1% FA, phase B: ACN with 0.1% FA, phase C: MeOH with 0.1% FA	Kinetex PFP core-shell column (10 mm × 2.1 mm, 1.7 μm) and PFP security guard ultra cartridge	THCCOOH-d3, THCCCOH-glu-d3 (and other ISs for different compounds)	LOQ: 10 ng/mL for THCCOOH-glu, 1 ng/mL for THCCOOH	10–500 ng/mL for THCCOOH-glu, 1–500 ng/mL THCCOOH	Analysis of plasma samples spiked with analytes with the use of fully automated Concept 96 device		[44]
CBD, CBC and terpenes	10 mg infloresences CBD dominant chemotype	Pulverization	Vac–HS–SPME/Reg–HS–SPME (65 μm coating + 10 μm overcoating) 75 μm PDMS/DVB coating; three sampling temperatures were tested (80 °C, 90 °C, and 150 °C). Three sampling time were tested (5, 15, 39 min) under both pressured condition	GC-MS Helium used as the carrier gas at flow rate 1.0 mL/min	Columns (two types were tested): Mega-5 (95% methylpolysiloxane 5%-phenyl) conventional (30 m × 0,25 mm, 0,25 μm), flow rate: 1 mL/min; narrow bore ( 15 m × 0,18 mm, 0,18 μm), flow rate: 0.72 mL/min	–	–	–	Simultaneous analysis of terpenes and PCs in CBD-rich chemotype		[49]
Δ9-THC, CBD, CBN	Cannabis plants, contact traces (0.08–4.28 mg)	Traces was collected with a dry cotton swabs, extracted with 1 mL MeOH, sonicated for 15 min, centrifuged, and diluted with ultrapure water (1:20–250).	In valve-IT-SPME TRB-5 based capillary coating (15 cm × 0.320, 3 μm); 10 μL of sample solution was percolated through capilllary.	nanoLC coupled with DAD detector 190–400 nm (210 nm for monitoring) Gradient elution: Mobile phase A: water Mobile phase B: ACN flow rate 0.5 μL/min	Zorbax 300SB C_18_ column (50 mm × 0,075 mm, 3,5 μm)	–	LOD: 2 ng/mL, LOQ: 8 ng/mL for Δ9-THC; LOD: 5 ng/mL, LOQ: 15 ng/mL for CBN, CBD	8–100 ng/mL for Δ9-THC, 15–100 ng/mL for CBN, CBD	Analysis of PCs in contact traces		[50]
CBD, Δ9-THC, Δ8-THC, CBN, CBG, CBC, CBV	10 mg plant material (stems, buds, flowers, leaves etc.) with different Δ9-THC and CBD content	–	HHS-SPME-GC/MS 100 μm PDMS fiber coating, incubation temperature: 140 °C, extraction: 150 s, desorption: 30 s, in GC injection port	GC-MS	Restek Rxi 35sil-M3 column ( 15 m × 0,25 mm, 0,25 μm)	–	–	–	Developing analytical platform of HHS-SPME extraction combined with machine learning for distinguishing cannabis sativa varieties		[51]
Δ9-THC, CBD	Human plasma (300 μL)	Samples were diluted with 650 μL of 10 mM acetate buffer (pH = 5).	SPME with magnetic restricted carbon nanotubes coated by bovine serum albumin, extraction: 5 min, desorption: 1 min with 200 μL ACN	UHPLC-MS/MS Gradient mode, phase A: 5 mM ammonium formate solution with 0.1% FA phase B: ACN flow rate: 0.3 mL/min 40 °C	Kinetex C_18_ column (100 mm × 2.1 mm, 1.7 μm)	Δ9-THC-d3	LOQ: 10 ng/mL for Δ9-THC and CBD	10–300 ng/mL		Analysis of plasma samples obtained from volunteers after single CBD intaking sessions and from chronic cannabis smokers	[54]
Δ9-THC, CBD, CBN, CBG, CBC, THCV, Δ8-THC	Various types of buccal swabs (5 mg)	Sample vial was incubated in 150 °C for 5 min with agitation.	HS-SPME with 100 μm PDMS coating, extraction time: 1 min, desorption: 0.5 min in GC injection port; derivatization with MSTFA during HS-SPME	GC-MS Helium as a carrier gas	Rxi-35 Sil MS column (15 m × 0.25 mm, 0.25 μm)	–	LOD: 0.2 ng/5 mg	–		Analysis of various types of buccal swabs spiked with analytes	[86]
Δ9-THC, CBN	Human hair (15 and 30 mg)	Hair samples were digested for 20 min at 80 °C with 1 mL of 1 M NaOH; the obtained solution was extracted two times with isooctane, and evaporated to dryness.	Derivatization with BSTFA during HS-SPME	GC-MS	–	Δ9-THC-d3, CBD-d3, CBN-d3	LOQ: 0,01 ng/mL	–		Analysis of 68 human hair samples (children, adolscentes, and cannabis user caregivers)	[87]
Δ9-THC, CBD, CBN	Human milk (50 μL)	pH was increased to 10 by addition of 1 M NaOH (10 μL) and bicarbonate buffer (940 μL); salting out of milk samples by addition of 0.25 mg of NaCl.	HS-SPME with 100 μm PDMS coating, extraction: 40 min at 70 °C, pH 10, desorption: in GC injection port at 250 °C	GC-MS, helium as the carrier gas at flow rate 0.6 mL/min	HP-5MS column (30 m × 0.25 mm, 0.1 μm)	THC-d3 CBD-d3 CBN-d3	LOD: 10 ng/mL, LOQ: 20 ng/mL for Δ9-THC, CBD, CBN	20–200 ng/mL	Analysis of 109 human milk samples		[89]

Δ8-THC: Δ8-tetrahydrocannabinol; Δ9-THC: Δ9-tetrahydrocannabinol; ACN: acetonitrile; BSTFA: N,O-Bis(trimethylsilyl)trifluoroacetamide; C_18_: octadecyl; CBC: cannabichromene; CBD: cannabidiol; CBG: cannabigerol; CBN: cannabinol; CBV: cannabidivarine; DI-SPME: direct immersion solid-phase microextraction; DVB: divinylbenzene; FA: formic acid; GC-MS: gas chromatography-mass spectrometry; IT-SPME: in-tube soli-phase microextraction; MeOH: methanol; OF: oral fluid; MSTFA: *N*-Methyl-*N*-trimethylsilyl-trifluoroacetamide; HHS-SPME: heated headspace solid-phase microextraction; HLB: hydrophilic-lipophilic balanced; HS-SPME: headspace solid-phase microextraction; LC: liquid chromatography; LLOQ: lower limit of quantification; LOD: limit of detection; LOQ: limit of quantification; PBS: phosphate buffered saline; PCs: phytocannabinoids; PDMS: polydimethylsiloxane; PFP: pentafluorophenyl; PPt: protein precipitation; SPME: solid phase microextraction; TF-SPME: thin film solid-phase microextraction; THC-COOH: 11-Nor-9-carboxy-Δ9-tetrahydrocannabinol; THCCOOH-glu: 11-nor-Δ9-tetrahydrocannabinol-9-carboxylic acid glucuronide; THC-OH: 11-hydroxy-Δ9-tetrahydrocannabinol; THCV: tetrahydrocannabivarine; UHPLC-MS/MS: ultra-high-performance liquid chromatography-tandem mass spectrometry; Vac–HS–SPME: vacuum-assisted headspace solid-phase microextraction.

Although easy to collect, urine samples may generate intense matrix effects, which can interfere with target analytes; therefore, sensitive and specific methods with MS detection should be implemented alongside advanced extraction procedures to monitor the level of PCs and their metabolites in this matrix. Boyacı et al. [43] proposed an approach consisting of thin-film SPME (TF-SPME) followed by LC-MS/MS for the simultaneous quantification of 110 compounds listed as prohibited substances by the WADA and their metabolites. Ten classes of prohibited compounds with different physicochemical properties were analyzed in this study, including PCs (Δ9-THC, CBD, CBN) and one Δ9-THC metabolite (THCCOOH). In the method-optimization stage, the authors tested four different types of coatings (C_18_, mixed-mode (C_18_-benzenesulfonic acid), phenylboronic acid (PBA), and PS-DVB) to determine which one provided the best extraction efficiency and lowest carryover effects. Although mixed-mode coating provided the best results for cannabinoids, ultimately C_18_ blades were chosen due to optimum recoveries and the lowest carryover effect of this coating for the extraction of all analytes from urine samples. In addition to determination of free substances, the proposed methodology was also able to extract the glucuronated forms of analytes, including THCCOOH-glu. It was observed that due to higher polarity of glucuronidated forms, their extraction was about 10% lower than free forms; however, no deconjugation reactions was needed in those analysis. Overall, despite the proposed protocol's speed and automation via the implementation of a Concept 96 device, it has yet to find a practical application. Significantly, the LOQ value obtained for Δ9-THC was higher than the minimum required performance levels (MRPL) set by WADA; thus, the proposed method cannot be applied to measure Δ9-THC concentrations in urine samples for antidoping testing. Elsewhere, IT-SPME was coupled with LS-MS/MS to quantify four PCs (Δ9-THC, THCV, CBD, CBN) and three Δ9-THC metabolites (THC-OH, THCCOOH and THCCOOH-glu) in human urine samples [41]. The IT-SPME column was prepared in the laboratory by packing LichroPrep RP-18 sorbent into a steel tube, as this coating provided the best extraction efficiency in comparison to the other sorbents (Luna C_8_, Luna C_18_ and LichroSorb RP-8) tested during the method-optimization stage. Since IT-SPME requires clean samples, a PPt step with acetonitrile (ACN) was added to the analytical protocol prior to performing extractions. Conversely, no hydrolysis was needed for the determination of glucuronides. The practical applicability of the proposed protocol was demonstrated by employing it for the analysis of cannabinoids in 20 urine samples collected from cannabis users. THCCOOH and THCCOOH-glu were detected in all analyzed samples. Concentration of THC-OH was only determined in one sample, while the remaining cannabinoids either went undetected or were detected below their LOQ values (Table 1). The detected levels of THCCOOH-glu ranged from < LOQ to 5,821 ng/mL, THCCOOH ranged from < LOQ to 1,885 ng/mL, and THC-OH was determined at the level of 40.24 ng/mL.

Human hair can accumulate compounds from the surrounding environment, as well as via systemic uptake. As such, it can often be challenging to differentiate the source of compounds in this matrix [87]. Cannabinoids accumulate in tissues easily due to their lipophilic character, and thus, they may be detectable long after consumption/external contact. While higher concentrations of THC-A (vs. neutral Δ9-THC) could be potentially a marker of hair external exposure to PCs, the precise monitoring of this compound may be hampered due to the time-consuming nature of hair sample preparation and thermal lability of THC-A during GC-based analysis [87]. Conversely, the presence of THCCOOH in human hair samples is a marker of systemic origin/consumption of PCs [87]. To select the best extraction and detection conditions for determination of PCs exposure, Moosmann et al. [87] compared three extraction methods—methanol extraction combined with LC-MS/MS for monitoring THC-A, Δ9-THC and CBN, alkaline hydrolysis combined with GC-MS/MS, and alkaline hydrolysis combined with SPME-GC-MS for monitoring Δ9-THC and CBN in hair samples obtained from parents who smoked *Cannabis*, along with hair samples obtained from their children and hair samples obtained from teenagers. However, in SPME-GC-MS protocol, only Δ9-THC and CBN were analyzed. Based on the obtained results, it was concluded that the use of alkaline hydrolysis in sample preparation step and other analytical conditions, such as high temperatures during the GC-MS/MS analysis led to artificially elevated levels of Δ9-THC. This was mainly related to the degradation of THC-A to Δ9-THC, Δ9-THC to CBN, and CBN to further oxidation products; thus, the determination of THC-A concentrations was not possible when this protocol was used.

Sweat is considered an alternative matrix to plasma in forensic studies, but the determination of PCs in this matrix only yield semi-quantitative results due to challenges associated with measuring the volume of collected fluid [35]. Despite this limitation, sweat analysis can still be useful for roadside screening. An HS-SPME-GC-MS method was proposed as a tool for preliminary drug control by Gentili et al. [35], who applied their method to determine 11 different recreational drugs, including Δ9-THC, in 66 real samples collected with a DrugWipe 5 A sweat-screening device. In this study, Δ9-THC was the only compound extracted directly into the alkaline environment without acidic hydrolysis, and measured concentrations of Δ9-THC ranged from 0.5 to 92.5 ng/pad. Based on their findings, the authors highlighted the need to verify the results of IA tests, as 7 samples returned false-positive results for the presence of Δ9-THC (Table 1).

The consumption of *Cannabis sativa* flower and cannabis-based products for recreational or medical purposes has also been observed among breastfeeding mothers. Δ9-THC is excreted into breast milk in amounts that could be clinically significant, as this compound remains detectable in breast milk for up to 6 weeks after *Cannabis* use. In addition, findings have shown that undetectable levels of Δ9-THC and its metabolites in plasma samples may not reflect their levels in breast milk [88]. Therefore, protocols enabling the direct monitoring of PCs in breast milk would be beneficial, as the detection of these compounds could help to prevent any life-threatening effects in breastfed infants [89]. The use of human breast milk as an analytical matrix can be challenging due to its high content of lipids, sugars, and proteins, as well as inter-individual variations in the concentration of these compounds [89]. These characteristics may result in significant matrix effects, which can prevent the efficient isolation and accurate analysis of the analytes under study. To overcome the drawbacks of this sample matrix, Silveira et al. [89] applied HS-SPME using 100 μm PDMS-coated fibers to isolate Δ9-THC, CBD, and CBN from 109 real human breast milk samples. In this study, a pH of 10 was selected as optimal based the results reported in previous studies, and natrium chloride (NaCl) was added to the sample to increase the extraction of analytes in HS mode. Overall, Δ9-THC was quantified in two samples with concentration level of 20 and 31 ng/mL, respectively, whereas CBD was detected below the LOQ value in one sample (Table 1).

Overall, SPME has been applied for the isolation of PCs from both conventional (plasma, urine) and non-conventional (OF, hair, sweat, breast milk) biological matrices. When handling plasma and urine samples, additional steps such as PPt, sample dilution, and/or pH adjustment can be implemented prior to SPME to reduce the risk of matrix effects and improve the overall method performance. Furthermore, in order to enhance the selectivity of the SPME coating during the isolation of PCs from complex matrices, various novel extraction phases have been synthesized and demonstrated satisfactory extraction efficiency of the analytes. It should also be highlighted that for urine samples, no hydrolysis was necessary prior to SPME extraction of the main PCs and their metabolites.

Regarding non-conventional matrices, OF was most frequently chosen for the determination of Δ9-THC, CBD, and CBN using SPME-GC-MS methods. Other alternative matrices used in forensic medicine studies to monitor PCs required special analytical protocols when employing SPME extraction. For instance, collecting sweat is not easy, and only semi-quantitative determination can be performed, although it allows the detection of analytes at low molecular concentrations. As PCs are labile compounds, additional sample handling may have a negative influence on the final results. Further sample manipulation, such as analyte derivatization or hair sample digestion, might enhance the method's sensitivity but could also lead to partial decarboxylation of acidic forms of cannabinoids as well as degradation and conversion of neutral forms of PCs into inactive compounds. Additionally, on-line extraction using IT-SPME protocols can offer advantages in terms of shorter analysis times, but it may sometimes require additional steps such as precipitation of proteins present in the sample matrix. On the other hand, SPME can be advantageous when dealing with very complex matrices, such as breast milk, since it prevents adsorption of large molecules, including proteins and sugars. Lastly, when designing new SPME-based protocols, it is essential to consider the risk of thermal degradation of PCs in the GC injection port or during the heating process in the HS extraction.

In summary, to overcome the drawbacks of traditional extraction techniques and also other approaches used in the analysis of PCs in complex matrices, SPME technology can be implemented as it facilitates precise monitoring of multiple compounds present at different concentration levels in the samples in a short analysis time and with no or minimum consumption of organic solvents. Recent advancements in the SPME technology applied in the analysis of major and minor PCs, including those present at trace amounts in plant material and animal/human matrices are summarized in this review. The application of different SPME devices with commercially available and in-lab made extraction phases has been described along with potential application of developed SPME-based protocols for the analysis of the level of particular PCs within forensic medicine, toxicological and pharmacological studies.

## 5 ECs—chemical structures, metabolism, and mechanisms of action in humans

In contrast to other types of neurotransmitters, ECs are mainly synthesized in the postsynaptic neurons and act as retrograde neurotransmitters. Once released into the synaptic cleft, they bind to the CB1 receptors in the presynaptic membrane and inhibit the release of other neurotransmitters, such as noradrenaline and serotonin. Another distinguishing feature of ECs is that, unlike other neurotransmitters, they are synthesized “on demand” and immediately metabolized to different compounds, including arachidonic acid (AA) instead of being stored [11]. Within the CNS, ECs play a role in thermoregulation, appetite control, memory processes, and mood, in addition to their psychoactive activity. ECs differ in their structure, potency, and affinity for cannabinoid receptors, which are membrane-coupled metabotropic receptors that contain the highest concentrations of CB1 in the CNS. Outside the CNS, CB1 and CB2 receptors are also found in the digestive tract, adipose tissue, and lungs. The ECS and its receptors have been the subject of intense research into the neurodegeneration that characterizes the development of Parkinson's disease (PD) and Alzheimer's disease (AD). Findings have shown that the overexpression of cannabinoid receptors creates a neuroprotective effect against PD and reduces neuroinflammation in AD, while Δ9-THC and CBD have also been shown to have neuroprotective effects in PD and AD animal models [90].

**Fig. 4:**
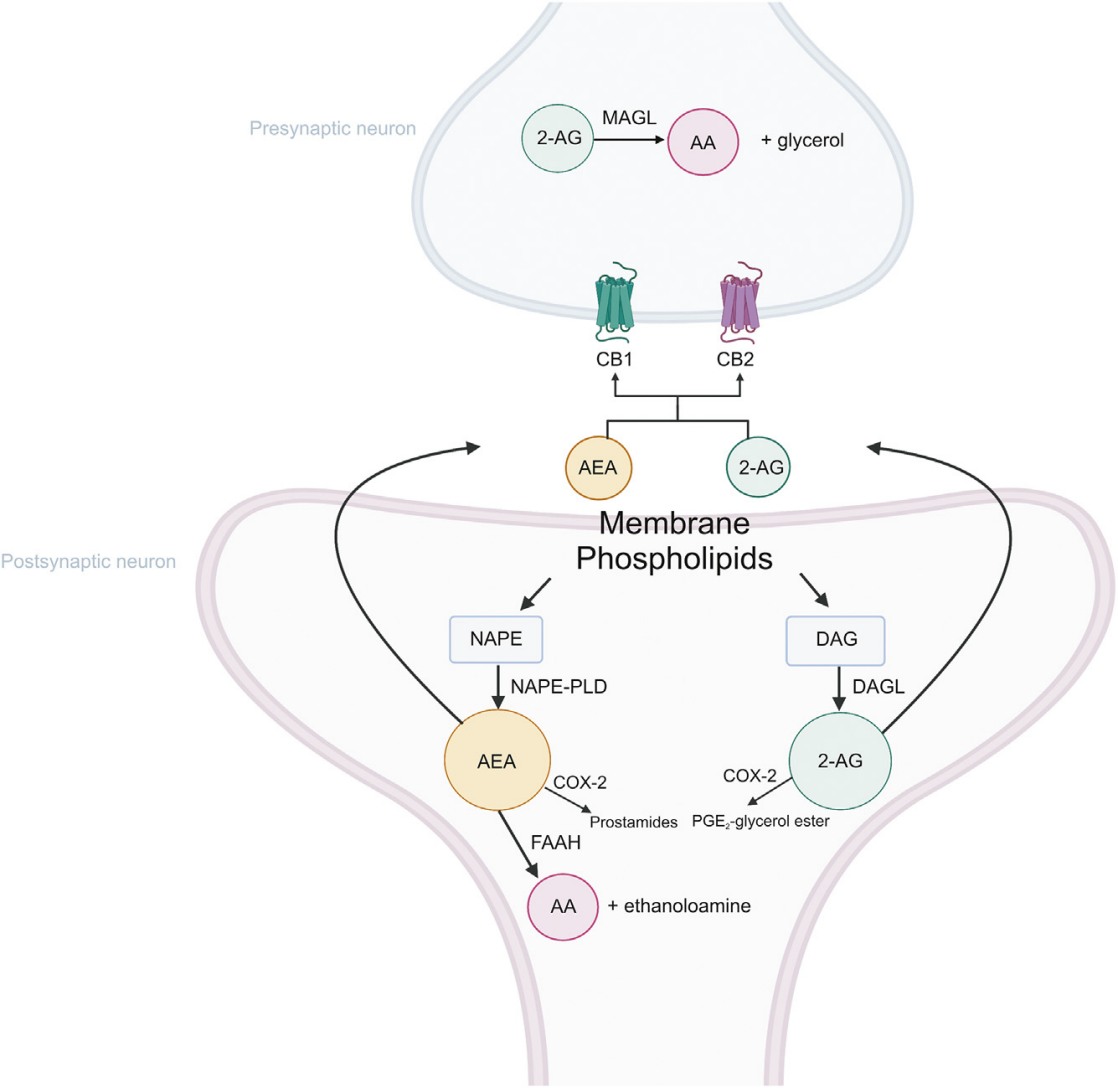
A simplified schematic pathways of endocannabinoids synthesis and degradation. AEA: anandamide; 2-AG: 2-Arachidonoylglycerol; NAPE: *N*-acylphosphatidylethanolamide; NAPE-PLD: *N*-acylphosphatidylethanolamide-phospholipase D; DAG: diacylglycerol; DAGL: diacylglycerol lipase; FAAH: fatty acid amidohydrolase; MAGL: monoacylglycerol lipase; AA: arachidonic acid; COX-2: cyclooxygenase 2; CB1: cannabinoid receptor type 1; CB2: cannabinoid receptor type 2. Figure is created with BioRender.com.

ECs are neurotransmitters that possess lipid structures and are derived from omega-6 polyunsaturated fatty acids. ECs are divided into three families: *N*-acylethanolamides (e.g., AEA), *N*-acyldopamines (e.g., *N*-arachidonoyl dopamine, NADA), and arachidonic acid derivatives (e.g., 2-AG and 2-arachidonoyl glycerol ether, 2-AGe). The main enzymes participating in their synthesis are *N*-acylphosphatidylethanolamine-phospholipase D hydrolase (NAPE-PLD), which catalyzes the synthesis of AEA, and diacylglycerol lipase (DAGL), which catalyzes the biosynthesis of 2-AG (Fig. 4). Once released from the receptors, ECs are rapidly metabolized by fatty acid amide hydrolase (FAAH) and monoacylglycerol lipase (MAGL) into ethanolamine, glycerol, AA, and other fatty acids [11]. AEA and other *N*-acylethanolamines are mainly metabolized by FAAH, while 2-AG and related compounds are degraded by MAGL and, to a lesser extent, FAAH. In addition, some AA oxidizing enzymes, such as cyclooxygenase 2 (COX2) [91] and lipoxygenases (LOX), metabolize ECs to produce prostaglandin glyceryl esters, prostaglandin ethanolamides, and eicosanoids, among other inflammatory molecules [91,92].

## 6 Determination of ECs in complex matrices

To date, levels of ECs have been determined in a variety of biological samples; however, blood and its fractions remain the most commonly used matrix for the monitoring of particular ECs [[27], [28], [29], [30],^93^], which are sometimes referred to as “circulating ECs” [1,94]. In addition, researchers have also employed other matrices, including cerebrospinal fluid [95], brain [10,29,52,96], hair [28,30,[97], [98], [99]], liver [100], and saliva [30], for trace determination of selected ECs. While most of these studies focused on the analysis of AEA and 2-AG, protocols for simultaneous determination of different ECs were also reported. Despite several studies on the ECS, little is known about it and the role of ECs in humans, as the analysis of these compounds is challenging due to their presence at trace levels (e.g., AEA) in biological samples, strong hydrophobicity and high protein binding properties [96,101]. Hence, the determination of such metabolites requires very efficient sample-preparation protocols and highly sensitive analytical instrumentation [9,29].

ECs are characterized by their presence at a wide range of concentrations (e.g., 2-AG is present at levels 1,000 times higher compared to AEA), low stability in samples, and absorption to plastic equipment during sample handling [29,97]. In addition, enzymatic degradation, prolonged sample preparation and sample storage are also commonly reported problems that can affect the obtained results [93]. With regards to the isolation of ECs from biological samples, the literature contains numerous reports of typical LLE-based protocols [10,28,29,96,101] and also SPE-based protocols [93,98,99]. Furthermore, the use of a PPt [1], PPt combined with LLE or SPE, and LLE combined with SPE [26,30,102] has also been applied. In order to overcome the drawbacks of traditional extraction protocols, the miniaturization of sample-preparation steps and development of on-line systems have become effective approaches that significantly shorten analysis times and enhance the precision and accuracy of methods applied to monitor the levels of particular ECs in biological samples.

### 6.1 The application of SPME for the extraction of ECs from animal/human matrices

**Fig. 5:**
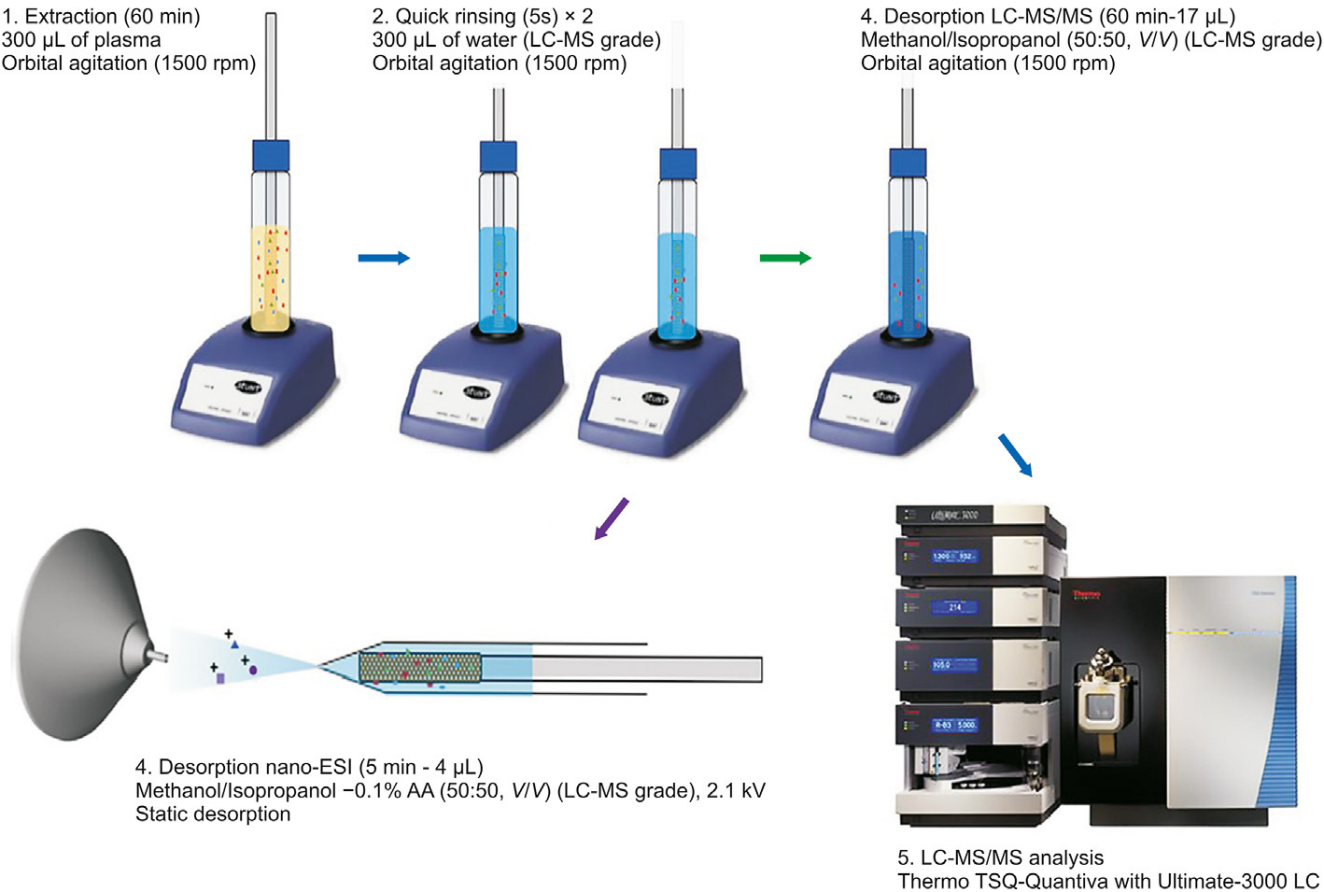
Analytical workflow of two solid-phase microextraction (SPME)-based methods (SPME-UHPLC-MS/MS and SPME-nano-ESI-MS/MS). Reprinted with permission from Ref. [27]. UHPLC-MS/MS: ultra-high-performance liquid chromatography-tandem mass spectrometry; nano-ESI-MS/MS: nano-electrospray ionization-tandem mass spectrometry.

The introduction of SPME technology to the bioanalysis of ECs has created new possibilities for analytical protocols by enabling shorter analysis times, eliminating errors related to sample handling, and allowing the isolation of unstable compounds present at trace levels in samples. Since 2019, protocols utilizing typical SPME workflows, modified IT-SPME, and in vivo SPME sampling have been employed for the determination of ECs in plasma samples and brain tissue (Table 2). In one study, Acquaro et al. [27] developed an SPME-based procedure that enabled the efficient isolation of two circulating cannabinoids: AEA and 2-AG. The authors tested various bio-SPME fibers (C_18_, triacontyl (C_30_), and HLB) and two analytical methods (UHPLC-MS/MS and nano-ESI-MS/MS) to identify the best conditions for the determination of ECs in human plasma. While the findings indicated that the HLB fibers provided best extraction efficiency, the high protein binding of the ECs necessitated matrix modification with guanidine hydrochloride and ACN to release bound ECs from the plasma proteins prior to extraction. The addition of this matrix-modification step enabled a 4.7- and 8.1-fold increase in the free concentrations of AEA and 2-AG, respectively. In addition, the use of a C_18_ superficially porous particle (1.7 μm) column facilitated the separation of 2-AG and 1-AG isomers possessing the same molecular mass but slightly different polarities. In the next stage of this study, two instrumental methods were developed for the precise analysis of both ECs. The SPME-UHPLC-MS/MS method required 60 min for desorption and 10 min for chromatographic analysis; conversely, in the nano-ESI workflow, a 5 min desorption was directly performed into the emitters, followed by 30 s for analyte separation and detection (Fig. 5) [27]. Ultimately, the SPME-UHPLC-MS/MS method provided better LOQ values for both analytes and was therefore proposed for use in further experiments aimed at monitoring EC levels in plasma samples. In another study, a wall-coated open tubular capillary column with polymeric ionic liquids (PILs) for on-line IT-SPME was coupled with UHPLC-MS/MS system to monitor AEA and 2-AG in plasma samples [53]. Selective PILs were synthetized from ionic liquids, namely, 1-vinyl-3-hexylimidazolium chloride ([VC_6_IM][Cl]), 1-vinyl-3-hexadecylimidazolium bromide ([VC_16_IM][Br]), and 1,10-di (3-vinylimidazolium) decane dibromide ([(VIM)_2_C_10_]2 [Br]) via in-situ thermal-initiated polymerization in a fused silica capillary column for IT-SPME. Among the evaluated sorbents, the PIL-based capillary with [VC_16_IM][Br]/[(VIM)_2_C_10_]2 [Br] provided the best extraction performance for both analytes, and it could be reused for more than ninety extractions without significant changes in the efficiency of the obtained results. Furthermore, the findings indicated that the chemically bonded and cross-linked PIL-based sorbent phase possessed high chemical and mechanical stability (Table 2). The lowest reproducibility for 2-AG was observed at a pH of 9 compared to pHs of 7 and 4, which can be explained by the spontaneous isomerization of 2-AG into 1-arachidonoyl-glycerol (1-AG) under some experimental conditions (e.g., high pH values in aqueous solutions). Hence, under optimized conditions, the concentration of 2-AG was quantified as the sum of the individual 2-AG and 1-AG peaks. Overall, the proposed IT-SPME-UHPLC-MS/MS method was verified by applying it to analyze AEA and 2-AG in real human plasma samples obtained from patients with PD. The mean levels of AEA and 2-AG detected by this method were 0.22 ng/mL and 0.29 ng/mL, respectively; however, the measured levels of 2-AG were much lower compared to those reported in other studies monitoring its concentration in regions of the brain.

**Table 2:** The overview of analytical approaches utilizing SPME-based techniques along with instrumental methods applied in the analysis of ECs in biological matrices.

Analyte(s)	Sample type and volume	Sample preparation		Instrumental analysis		Internal standard(s)	LOD/LOQ	Linearity	Application	Refs.
		Pretreatment	Extraction	Chromatographic separation	Detection					
AEA, 2-AG, 1-AG	Human plasma (300 μL)	PPt with 200 μL of matrix modiﬁer solution	SPME with HLB coating, extraction: 60 min, desorption: 60 min in MeOH:IPA (50:50, *V/V*) for SPME-UHPLC-MS/MS, or 5 min in MeOH:IPA (50:50, *V/V*) with 0.1% acetic acid for Bio-SPME-nano-ESI-MS/MS	UHPLC-MS/MS gradient mode: phase A: water with 0.1% of acetic acid, phase B: ACN:IPA (90:10, *V/V*), flow rate: 0.3 mL/min at 40 °C	UHPLC-MS/MS: CORTECS C_18_ superficially porous column (100 mm × 2.1 mm, 1.6 μm), and CORTECS C_18_ superficially porous guard column (5 mm × 2.1 mm, 1.6 μm)	AEA-d4, 2-AG-d5, 1-AG-d5	LOQ: 50 ng/mL for AEA and 2-AG	SPME-UHPLC-MS/MS: 1–200 ng/mL, SPME-nano-ESI-MS/MS: 50–800 ng/mL	Analysis of plasma samples spiked with the analytes	[27]
AEA, 2-AG	1% agarose gel and rat brain	–	In vivo SPME with 1.5 mm length RP-amide-C_16_ coating, extraction: 30 min, desorption: 45 min in ACN:water (72:25, *V/V*)	Gradient mode: phase A: 0.01 M ammonium acetate, phase B: ACN, flow rate: 0.3 mL/min at 40 °C	BEH C_18_ column (0.21 cm × 5 cm, 1.7 μm)	AEA-d4, 2-AG-d5	LLOQ: 0.05 ng/mL	0.05–50 ng/mL	In vivo brain sampling of BST and PH regions in male Sprogue–Dawley rats (loud noise stress group and no noise control group)	[52]
AEA, 2-AG	Human plasma (400 μL)	PPt with 800 μL of ACN; the supernatant was dried and then reconstituted with 120 μL of 90:10 (*V/V*) mixture of 5 mM ammonium acetate (pH 7.0) aqueous solution and ACN.	On-line IT-SPME with PIL-based (10.0 cm × 0.53 mm) capillary column; 80 μL sample solution was percolated through the PIL-based capillary using water (solvent A); flow rate: 0.1 mL/min	Mobile phase used for desorption step: 0.5% FA in water (solvent B) and ACN with 0.5% FA (solvent C) at 30:70 (*V/V*) ratio for 2 min, flow rate: 0.1 mL/min at 40 °C	Core-shell Kinetex C_18_ column (100 mm × 2.1 mm, 1.7 μm)	AEA-d4, 2-AG-d5	LLOQ: 0.1 ng/mL for AEA, 0.05 ng/mL for 2-AG	0.1–100 ng/mL for AEA, 0.05–100 ng/mL for 2-AG	Analysis of plasma samples obtained from six patients with PD	[53]
AEA, 2-AG	Brain homogenate (150 μL)	Homogenization with 0.1 mol/L FA solution; PPt with 150 μL of ACN and 100 μL of 5 mol/L ammonium formate solution; the upper organic phase was collected, dried and reconstituted with 50 μL of water:ACN (30:70, *V/V*).	IT-SPME with RAM phase; 10 μL of sample solution was percolated through the RAM microtube with mobile phase consisting of water:ACN (80:20, *V/V*); extraction time: 3 min, flow rate: 0.2 μL/min	Mobile phase used for desorption step: 0.5% FA aqueous solution:ACN ( 30:70, *V/V*) for 3 min, flow rate: 0.2 μL/min	–	AEA-d4, 2-AG-d5	LOQ: 6 ng/mL for AEA, 10 ng/mL for 2-AG	6–30 ng/mL for AEA, 10–100 ng/mL for 2-AG	Analysis of 10 brain samples from Wistar male rats lesioned with 6-hydroxydopamine; two striatum tissue samples were obtained from each animal: one ipsilateral to the lesion and another contralateral to the lesion.	[103]
AEA, 2-AG, NADA, 2-AGe	PBS (1 mL) and intact rat brain	–	SPME with 4 mm length C_18_ coating, extraction: 30 min, desorption: 30 min in MeOH:IPA (50:50, *V/V*)	Gradient mode: phase A: water with 0.1% FA, phase B: ACN with 0.1%FA, flow rate: 0.3 mL/min at 30 °C	Kinetex XB-C_18_ column (100 mm × 2.1 mm, 2.6 μm)	AEA-d11	LOD: 0.4 ng/mL for AEA, 5 ng/mL for 2-AG, 1 ng/mL for NADA and 2-AGe	5–150 ng/mL for AEA, NADA and 2-AGe, 50–1,500 ng/mL for 2-AG	Analysis of intact (non-homogenized) brain structures (cortex, cerebellum and striatum) of Wistar Han female and male rats at different stages of development (1 month old, 3 months old, and 24 months old)	[104]

1-AG: 1-arachidonoyl glycerol; 2-AG: 2-arachidonoyl glycerol; 2-AGe: arachidonoyl glycerol ether; ACN: acetonitrile; AEA: *N*-arachidonoyl ethanolamide; BEH: ethylene bridged hybrid; BST: stria terminalis; FA: formic acid; HLB: hydrophilic-lipophilic balanced; IPA: isopropyl alcohol; IT-SPME: in-tube solid phase microextraction; LLOQ: lowest limit of quantification; LOD: limit of detection; LOQ: limit of quantification; MeOH: methanol; NADA: *N*-arachidonoyl dopamine; PBS: phosphate buffered saline; PD: Parkinson's disease; PH: posterior hypothalamus; PIL: polymeric ionic liquid; PPt: protein precipitation; RAM: phase-restricted access material phase; SPME: solid-phase microextraction; UHPLC-MS/MS: ultra-high-performance liquid chromatography-tandem mass spectrometry.

Interestingly, an approach directly coupling IT-SPME to an MS/MS system was developed for the determination of AEA and 2-AG in homogenized rat brain samples. The IT-SPME device used in this work consisted of a microtube of restricted access material (RAM) with a hydrophilic diol external surface and a hydrophobic octyl inner surface [103]. Prior to SPME, the samples were subjected to PPt with ACN. The authors determined the endogenous concentrations of AEA and 2-AG in rat brain striatum following the administration of the neurotoxin, 6-hydroxydopamine (6-OHDA), into the nigrostriatal pathway to cause the destruction of dopaminergic neurons that occurs during the development of PD. The results demonstrated that concentrations of both ECs differed in the ipsilateral and contralateral regions of the striatum, and that the optimized IT-SPME-MS/MS protocol enabled the precise determination of levels of both ECs in brain samples. In particular, the findings showed that concentrations of AEA were higher in the striatum tissue ipsilateral to the lesion than in the striatum tissue contralateral to the lesion (the concentrations ranged from 11.03 to 13.86 ng/mL and from 10.34 to 11.78 ng/mL, respectively); in contrast, the concentrations of 2-AG in the striatum tissue ipsilateral to the lesion were lower than in the striatum tissue contralateral to the lesion (the concentrations ranged from 2.08 to 2.49 μg/mL and from 3.95 to 4.42 μg/mL, respectively). Aslam et al. [52] developed an in vivo SPME protocol with LC-MS analysis to monitor how stress (loud noise) affected the levels of AEA and 2-AG in the brain structures of living rats. To this end, the authors prepared SPME fibers coated with a new extraction phase composed of RP-amide-C_16_-silica particles, which were directly introduced into two brain regions in the rats to isolate the two ECs, followed by LC-MS analysis. For SPME method optimization, 1% agarose gel mixed with brain homogenate has been proposed as a surrogate matrix [52]; however, this approach can result in the thermal degradation of labile ECs, such as NADA. In vivo sampling was performed on 24 male rats by inserting the SPME fibers into the bed nucleus of the stria terminalis (BST) and posterior hypothalamus (PH) on day 1 and day 11 after repeated exposure to loud noise (experimental group) or no noise (control group). The findings showed that the concentrations of ECs in the BST and PH regions ranged between 0.3 and 40 ng/mL and differed between the control and experimental groups. Recently, Roszkowska et al. [104] coupled a modern SPME-based methodology to LC-MS/MS to monitor four ECs (AEA, 2-AG, NADA, and 2-AGe) in three intact (non-homogenized) brain structures in female and male rats at different stages of development (young, adult, and old). After optimizing the microextraction conditions, 4-mm-long biocompatible C_18_ probes were introduced into the cerebellums, cortexes, and striata of the studied rats. Several factors were considered in an attempt to enhance analyte stability and method performance in order to facilitate the in vivo quantification of trace and unstable ECs in the CNS. Specifically, the designed analytical protocol sought to develop a more detailed understanding of the role played by these compounds in the function of the studied brain structures. The proposed protocol successfully enabled the extraction and quantification of 2-AG and AEA from each brain region, with 2-AG being present at significantly higher levels compared to AEA. This result is consistent with those reported in other works. Significantly, this study marked the first documented extraction of two highly unstable ECs, NADA and 2-AGe, using SPME probes. The developed SPME-LC-MS/MS protocol facilitated the isolation of highly unstable endogenous compounds from intact tissue, confirming its suitability for monitoring even trace levels of ECs in complex biological matrices. In particular, this novel microsampling technique can enable the precise determination of the levels and distribution of ECs in different brain regions, even for in vivo applications.

## 7 Conclusions

Given the continued growth in the consumption of PCs (i.e., components of *Cannabis sativa* flowers) for medical and non-medical purposes, it is becoming increasingly important to develop and optimize analytical protocols that combine advanced extraction techniques and sensitive instrumental analysis for the efficient isolation and determination of these compounds in complex matrices. Such protocols could be invaluable in routine forensic medicine and toxicological analysis, as well as in studies examining the role of the ECS in physiological conditions and the development of diseases such as PD. SPME is a fast, simple, and green sample-preparation technique that has shown great potential for the determination of low-molecular-weight compounds in pharmaceutical and metabolomics studies, as it enables the isolation of a broad range of hydrophilic and hydrophobic molecules from biological fluids and intact (non-homogenized) tissues. SPME facilitates a significant reduction in matrix effects caused by ballast substances in the sample, which in turn ensures the production of clean extracts that can be directly subjected to analysis with sensitive instruments, such as MS. Over the last 10 years, different SPME-based protocols have been proposed for the isolation of PCs and ECs from complex matrices. To date, SPME has been implemented to isolate PCs from typical matrices used in bioanalysis, such as plasma and urine, as well as from less-conventional biological matrices, such as OF, hair, and breast milk. These analyses have mainly been performed in HS-SPME and DI-SPME modes, but optimized IT-SPME has also enabled the efficient isolation and monitoring of THC, CBD, and/or their metabolites in the aforementioned biological matrices. However, it should be emphasized that the use of HS-SPME can result in the degradation of neutral forms of PCs. In plant material, SPME has been applied to monitor Δ9-THC, CBD, CBN, and other PCs; nonetheless, we are unaware of any SPME protocol that has been developed for the determination of PCs in their acidic form. In the case of ECs (i.e., AEA and 2-AG), SPME devices with typical and novel coatings have been implemented to enable the precise monitoring of their circulation and to isolate them from brain samples. Although different SPME-based approaches have been proposed for the quantification of ECs in biological matrices, their determination in humans remains challenging due to their endogenous nature, high instability, and rapid degradation/biotransformation. Since cannabis-based medicines have become legal in many countries, the impact of their components—particularly how PCs influence ECS function and EC levels—has emerged as an important and developing area of research. The determinations of ECs and PCs requires advanced analytical techniques and, in this respect, SPME may be the ideal tool for analyzing the correlation between phyto- and endocannabinoids, as well as their therapeutic potential and their relationships in various clinical aspects, especially related to the functioning of the CNS. SPME's ability to enable precise monitoring of the composition and levels of particular PCs and ECs in plant and human matrices and its suitability for in vivo applications may help to develop a more robust understanding of the molecular cascade that follows cannabinoid treatment, especially in relation to the impact of PCs and ECs on the ECS. Such information could be the key in identifying drug targets for preventing and treating CNS diseases, such as neurodegenerative diseases.

## CRediT author statement

**Katarzyna Woźniczka:** Conceptualization, Resources, Data curation, Writing - Original draft preparation, Reviewing and Editing, Visualization; **Paweł Konieczyński:** Conceptualization, Resources, Data curation, Writing - Original draft preparation, Reviewing and Editing, Visualization; **Alina Plenis:** Conceptualization, Resources, Data curation, Writing - Original draft preparation, Reviewing and Editing, Visualization; **Tomasz Bączek:** Writing - Original draft preparation, Reviewing and Editing; **Anna Roszkowska:** Conceptualization, Resources, Data curation, Supervision, Writing - Original draft preparation, Reviewing and Editing, Visualization.

## Declaration of competing interest

The authors declare that there are no conflicts of interest.
